# Intramural Bowel Hematoma Presenting as Small Bowel Obstruction in a Patient on Low-Molecular-Weight Heparin

**DOI:** 10.1155/2018/8780121

**Published:** 2018-06-13

**Authors:** Beatrix Hyemin Choi, Michael Koeckert, Sandra Tomita

**Affiliations:** ^1^Division of Pediatric Surgery, Department of Surgery, NYU School of Medicine, Hassenfeld Children's Hospital at NYU Langone, New York, NY, USA; ^2^Department of Surgery, NYU School of Medicine, NYU Langone Medical Center, New York, NY, USA

## Abstract

There is increasing use of low-molecular-weight heparin (LMWH) for treatment of pediatric thromboembolic disease as it has been shown to be safe and effective. It has several advantages over unfractionated heparin, such as reduced need for monitoring, easier route of administration, decreased risk of heparin-induced thrombocytopenia, and lack of drug-drug interactions. Nevertheless, LMWH still poses a bleeding risk as with any anticoagulant therapy. We present the case of a 4-year-old boy who was placed on LMWH for a catheter-related deep venous thrombosis in the setting of intractable seizures and subsequently developed a small bowel obstruction secondary to a suspected intussusception. He underwent exploratory laparotomy and was found to have an intramural bowel hematoma. Prior to this bleed, the patient had been monitored daily, and his anti-Xa levels were found to be in the therapeutic range. This case highlights the need for a high index of suspicion for spontaneous bleeding even in the setting of therapeutic anti-Xa levels.

## 1. Introduction

Low-molecular-weight heparin (LMWH) has been increasingly used to treat thromboembolic disease in children as it has been shown to be as safe and effective as unfractionated heparin [1]. LMWH has several advantages over unfractionated heparin: reduced need for monitoring, subcutaneous rather than intravenous administration, reduced risk of heparin-induced thrombocytopenia (HIT), and lack of interaction with other drugs or diet [2]. As with any anticoagulant, the main risk of LMWH use is bleeding. We present the case of a boy on LMWH with anti-Xa levels within therapeutic range, who developed a small bowel intramural hematoma leading to bowel obstruction and partial resection.

## 2. Case

A 4-year-old boy was transferred to our pediatric intensive care unit from an outside hospital for further management of a persistent seizure disorder of unknown etiology. A right femoral triple lumen central venous line (CVL) had been placed prior to transfer. Five days after arrival, the patient began to exhibit increased swelling in his right lower extremity, and ultrasonography revealed a catheter-related, acute occlusive deep venous thrombosis in the right common femoral vein. He was started on LMWH (enoxaparin) at 1 mg/kg for a planned course of 3 months. Five days after initiating treatment, the CVL was removed. The patient had no personal or family history of thrombophilia or bleeding diathesis. His anti-Xa level, checked after the second dose, was within the therapeutic range.

**Figure 1:**
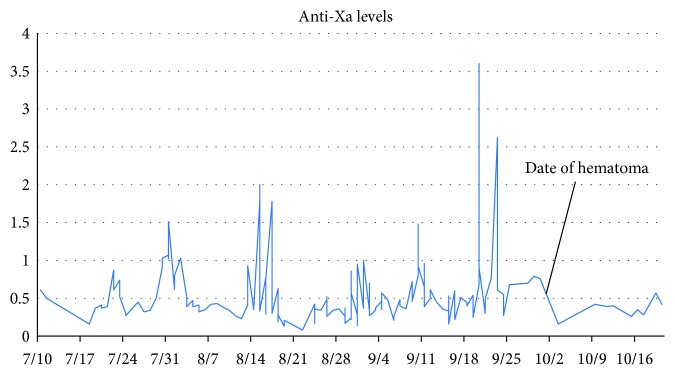
Anti-Xa levels of the patient throughout his hospital admission. LMWH doses are not included as they varied from day to day following the patient's lab results. For reference, the regimen used after September 7 was as follows: <0.35 units/mL: increased dose by 25%, repeat anti-Xa 4 hours after next dose. 0.35–0.49 units/mL: increased dose by 10%, repeat anti-Xa 4 hours after next dose. 0.5–0.59 units/mL: keep same dose, repeat anti-Xa next day. 0.6–1.0 units/mL: contact on call fellow for further recommendations. 1.0–1.5 units/mL: decrease dose by 20%, repeat level before next dose. 1.6–2 units/mL: hold dose for 3 h, then decrease dose by 30%. Repeat level before next dose, then 4 h after next dose. >2 units/mL: hold all doses until anti-Xa is 0.5 units/mL, then decrease dose by 40%. Repeat level before next dose, then q12h until anti-Xa is <0.5 units/mL.

His hospital course was complicated by multisystem organ failure in the setting of drug reaction with eosinophilia and systemic symptoms (DRESS) syndrome secondary to anticonvulsive therapy. One week after starting LMWH heparin, the patient experienced gross hematuria. The next day, the injection sites were noted to be slightly oozy, and, in the setting of his anti-Xa levels continuing to rise (0.87), LMWH heparin was held. He required continuous venovenous hemofiltration, during which time anticoagulation was switched to unfractionated heparin. After renal recovery, LMWH therapy was restarted at a lower dose (70% of original dose), but his anti-Xa levels continued to be labile and difficult to control. Eventually, a steady regimen was found with consistently stable and therapeutic anti-Xa levels (Figure 1).

**Figure 2:**
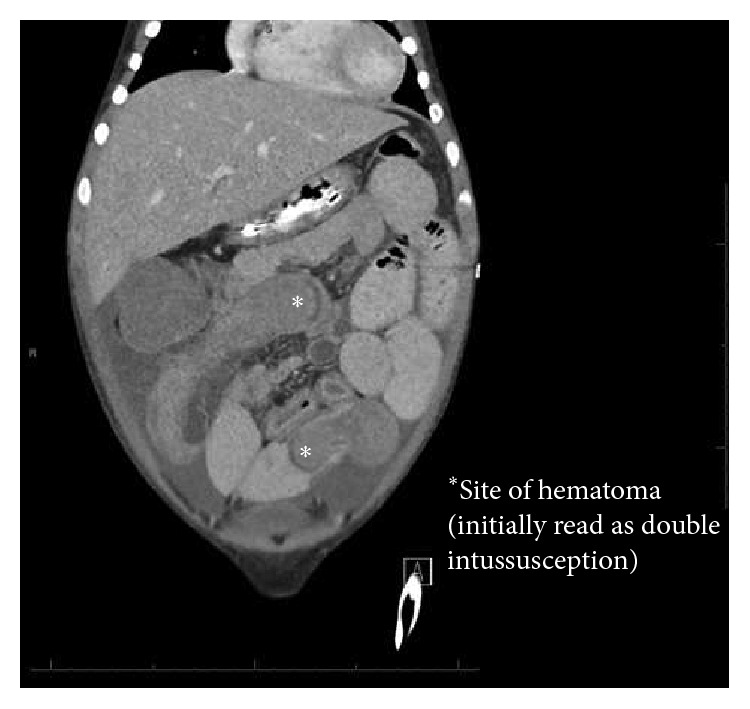
Coronal CT.

**Figure 3:**
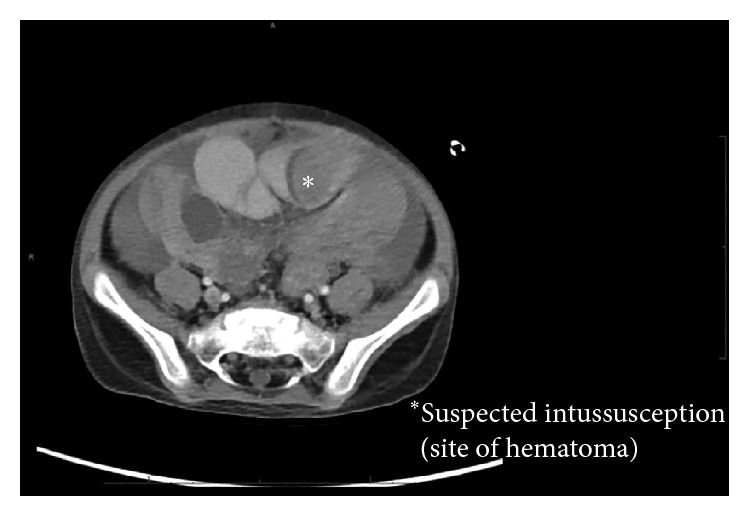
Axial CT.

On the 15th day of this regimen, however, he developed signs of bowel obstruction with new onset of copious bilious vomiting. An abdominal ultrasound found a small amount of fluid in the pelvis. A CT of the abdomen and pelvis showed a high-grade small bowel obstruction, with 2 areas of small bowel, suspicious for intussusception (Figures 2 and 3). The patient's hemoglobin was found to have dropped from 9.9 to 6.2.

**Figure 4:**
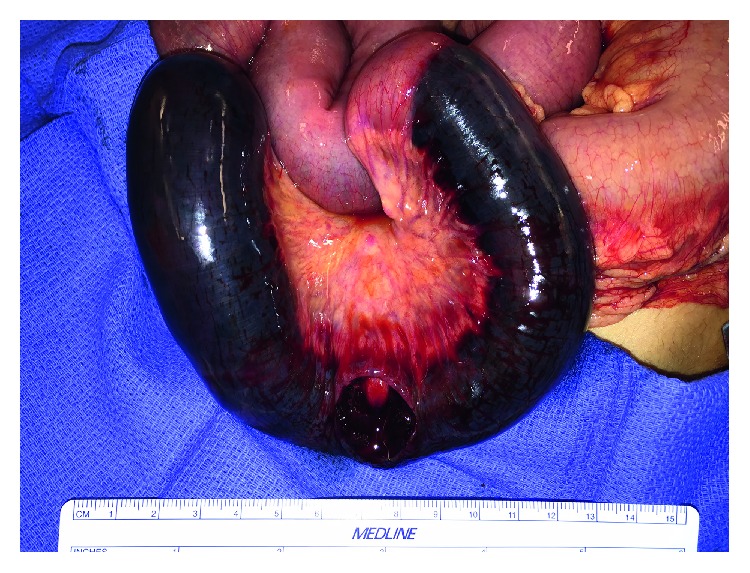
Intramural hematoma.

He was brought to the operating room for exploratory laparotomy. Intraoperatively, bloody ascites and multiple dilated loops of small bowel were found. Approximately 30 cm of the distal jejunum was found to be tense, heavy, firm, and discolored with a blue hue, with a hematoma that had dissected through the layers of the bowel (Figure 4). A serosal defect was found on the antimesenteric border of the involved bowel, likely causing the bloody ascites. As the bowel was severely compromised, a resection of the involved segment was performed.

Pathologic analysis of the resected bowel showed an extensive, 35 cm submucosal hematoma causing internal bulging of the mucosa and submucosa, causing attenuation and focal pressure necrosis of the muscularis propria. The serosa was noted to be diffusely purple-red, with a small ovoid defect that was presumably caused by the hematoma. At both ends of the resected bowel, there was evidence of the mucosa and submucosa bulging into the lumen of the bowel as a result of the hematoma.

The patient recovered from the operation in the pediatric ICU with no further episodes of emesis or signs of bowel obstruction. He was restarted on LMWH 5 days later and completed his 3-month course without any further signs of bleeding. He was discharged from the hospital 3 weeks later with resolution of his seizures to a rehabilitation facility, without any further complications of bleeding or bowel obstruction.

## 3. Discussion

LMWH has become the preferred agent for the prophylaxis and treatment of thromboembolic disease in children in whom venous access is difficult. It has been shown to be a safe and effective treatment in adults, with reduced incidence of complications such as HIT and osteoporosis. One of the main risks of LMWH, as with any anticoagulative therapy, is bleeding. While no adequately powered studies have assessed the rates of hemorrhage in children on LMWH, various studies have reported the incidence of major bleeding events from 3 to 9%, of which gastrointestinal bleeds make up a small fraction [1, 3–6].

Nontraumatic spontaneous intramural small bowel hemorrhage is a rare complication of anticoagulation therapy. The incidence in adults is reported to be 1 in 2500, with the jejunum being the most common site of a hematoma [7]; there are no studies reporting the incidence of spontaneous small bowel hemorrhage in children. To date, there are only two cases reporting a small bowel hematoma in a child receiving anticoagulation [8, 9]. In both cases, the children had developed thromboembolic disease necessitating anticoagulation with LMWH. In these cases, it was unclear whether LMWH dosing was a factor in the hemorrhage as anti-Xa levels were not monitored or infrequently monitored. Our patient developed hemorrhage despite closely monitored therapeutic anti-Xa levels. In hindsight, the patient's bleeding history on LMWH in conjunction with the rapid drop in hemoglobin are vital pieces of clinical history that should have raised the level of clinical suspicion for gastrointestinal bleeding rather than intussusception.

These cases demonstrate the importance of maintaining a high index of suspicion for gastrointestinal hemorrhage as a complication of LMWH therapy despite its demonstrated efficacy and safety in the adult population. Patients should be monitored closely with plasma anti-Xa levels and appropriate therapeutic doses. However, it is important to keep in mind that a therapeutic anti-Xa level in children does not necessarily equate to safety due to differences in plasma binding and clearance. This case further underscores the importance of clinical monitoring for a patient even with therapeutic anti-Xa levels, as gastrointestinal bleeds can develop rapidly and become life threatening.
